# Microwave-to-optical conversion with a gallium phosphide photonic crystal cavity

**DOI:** 10.1038/s41467-022-28670-5

**Published:** 2022-04-19

**Authors:** Simon Hönl, Youri Popoff, Daniele Caimi, Alberto Beccari, Tobias J. Kippenberg, Paul Seidler

**Affiliations:** 1 IBM Quantum, IBM Research Europe, Zurich, Säumerstrasse 4, CH-8803 Rüschlikon, Switzerland; 2 Integrated Systems Laboratory, Swiss Federal Institute of Technology Zurich (ETH Zürich), CH-8092 Zürich, Switzerland; 3 Institute of Physics, Swiss Federal Institute of Technology Lausanne (EPFL), CH-1015 Lausanne, Switzerland

**Keywords:** Photonic crystals, Nanophotonics and plasmonics, Optomechanics, Nanophotonics and plasmonics, Photonic crystals

## Abstract

Electrically actuated optomechanical resonators provide a route to quantum-coherent, bidirectional conversion of microwave and optical photons. Such devices could enable optical interconnection of quantum computers based on qubits operating at microwave frequencies. Here we present a platform for microwave-to-optical conversion comprising a photonic crystal cavity made of single-crystal, piezoelectric gallium phosphide integrated on pre-fabricated niobium circuits on an intrinsic silicon substrate. The devices exploit spatially extended, sideband-resolved mechanical breathing modes at ~3.2 GHz, with vacuum optomechanical coupling rates of up to *g*_0_/2*π* ≈ 300 kHz. The mechanical modes are driven by integrated microwave electrodes via the inverse piezoelectric effect. We estimate that the system could achieve an electromechanical coupling rate to a superconducting transmon qubit of ~200 kHz. Our work represents a decisive step towards integration of piezoelectro-optomechanical interfaces with superconducting quantum processors.

## Abstract (web summary)

A route to scalability for superconducting quantum computation is the modular approach, which however requires coherent microwave-to-optical conversion. Here the authors use gallium phosphide optomechanical crystal cavities for this task, exploiting their high refractive index and large OM coupling rate.

## Introduction

Recent years have seen the advent of quantum computers based on superconducting microwave circuits and their rapid development^1,2^ towards commercial systems that outperform classical computers. Due to their low energy, the microwave qubits are bound to the millikelvin environment of a dilution refrigerator, which presents a variety of challenges for building systems with large numbers of qubits. A particularly interesting approach to solving the scaling problem is networking of smaller machines via quantum-coherent interconnects^3–5^. Communication between quantum processing units at microwave frequencies must, however, be carried out through cryogenically cooled waveguides^6^ to avoid decoherence. An attractive alternative is to map the quantum state from the microwave domain to the optical domain, where information can be transmitted quantum coherently through low-loss optical fibers^7^. Numerous methods have been proposed for microwave-to-optical conversion^8–26^; the most efficient to date make use of an intermediary mechanical state^27–35^.

Great strides have been made in the field of cavity optomechanics^36^ during the past decade in demonstrating the building blocks needed for quantum-coherent optical interconnects, including the demonstration of quantum control of single-phonon states in mechanical modes at gigahertz frequencies with single photons^37^, the realization of optomechanically mediated quantum entanglement of separate mechanical systems^38^, and the conversion of a superconducting qubit excitation to an optical photon^39^. Progress has also been made in the manipulation of quantum states of a mechanical oscillator through interaction with a superconducting qubit^40–42^.

We introduce here a platform for microwave-optical conversion that combines a microwave circuit fabricated on an intrinsic silicon substrate with an optomechanical element made of single-crystal gallium phosphide (GaP), using direct wafer bonding. For the optical cavity, we employ a quasi-one-dimensional optomechanical crystal cavity^43^ with extended mechanical modes that permit electrical actuation remote from the optical mode to reduce losses. The extended modes nevertheless maintain substantial optomechanical coupling. Due to the relatively high index of refraction of GaP (3.05 at *λ*_*vac*_ = 1550 nm) and the consequent strong light confinement, GaP cavities have vacuum optomechanical coupling rates similar to those of devices made of silicon^44,45^ and significantly larger than those of lithium niobate^30^ or aluminum nitride^33^ devices. In terms of heating in pulsed experiments, GaP devices compare favorably to structures made of gallium arsenide and other piezoelectric materials^45,46^. Making use of the piezoelectric properties of GaP, we demonstrate at room temperature actuation of the mechanical modes of the optomechanical device with integrated electrodes and coherently transduce microwave signals to optical frequencies.

## Results

### Device design

**Fig. 1:**
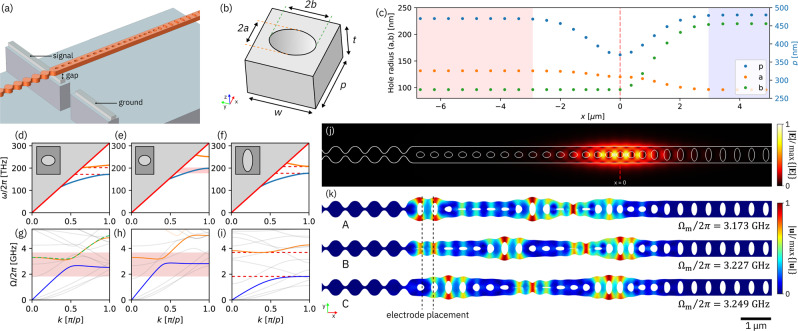
GaP optomechanical crystal cavity design. **a** Illustration of the cavity suspended over an electrode pair. **b** Schematic of unit cell (*t* = 300 nm, *w* = 550 nm). **c** Variation of unit-cell parameters as indicated in **b** along the cavity *x*-axis. The shading indicates the phononic waveguide (red), cavity (white), and input-mirror (blue) regions. **d**–**f** Photonic band structure of the unit cell for the phononic waveguide, cavity center, and input mirror, respectively. The light line is indicated in red. The red-shaded region in **e** is the intersection of the photonic bandgaps in **d** and **f**. **g**–**i** Phononic band structure of the unit cell ordered as in **d**–**f**. The mechanical breathing modes are indicated in blue and orange. The red shaded region in **g** and **h** corresponds to the phononic bandgap in **i**. For comparison, the upper breathing mode band for the unit cell at the cavity center is overlayed in **g** as a dashed green line scaled for the difference in period *p*. **j** FEM simulation of the localized optical mode. The color scale indicates the magnitude of the electric field ∣**E**∣. **k** FEM simulation of three extended mechanical breathing modes. The color scale indicates the magnitude of the mechanical displacement ∣**u**∣.

Our transducer consists of an asymmetric quasi-one-dimensional optomechanical crystal cavity made of GaP suspended over a niobium electrode at one end, with a second, coplanar niobium electrode displaced to one side, as illustrated in Fig. 1a. Laser light is coupled in and out of the device through a waveguide attached to it on the right side. The geometry of the optomechanical crystal cavity is adapted from the design introduced by Chan et al.^43,47^ and consists of a series of elliptical holes etched in a rectangular beam. The hole radii and pitch are, however, modified to create distributed mechanical modes extending between the center of the cavity and the electrode position^39^. Finite-element-method (FEM) simulations are employed to model both the photonic and phononic band structure, determine the intrinsic, radiation-limited optical quality factor, *Q*_0_, and estimate the vacuum optomechanical coupling rate, *g*_0_.

The design is composed of three sections (Fig. 1c). At the right end of the GaP beam (which is 300 nm thick and 550 nm wide), the unit cell is chosen to form a partially transparent photonic mirror with an optical bandgap for TE-polarized light between 177 and 208 THz, where the optical coupling rate to the cavity can be controlled by varying the number of holes. This section also has a bandgap for mechanical breathing modes between 1.82 and 3.69 GHz and thus serves as a phononic mirror as well. On the left side of the device, the unit-cell geometry is again chosen to create a photonic bandgap, this time between 172 and 202 THz, but now has a phonon dispersion that supports mechanical breathing modes within the phononic bandgap of the input mirror on the right side of the device. Between these two regions, in the central portion of the beam, the unit-cell dimensions are varied to produce a confined optical mode, with the variations on each side following Gaussian functions of width *σ* = 1 μm. The result is a cavity supporting a localized optical mode (Fig. 1j) at *ω*_o_/2*π* = 193.2 THz with *Q*_0_ = 1.8 × 10^6^. Crucially, the dispersion of the mechanical breathing modes is nearly identical at the center of the cavity and on the left side of the device (see the dashed green line in Fig. 1g), resulting in an impedance-matched phononic waveguide. The device is terminated on the left with a phononic reflector exhibiting a complete bandgap between 2.84 and 3.51 GHz, implemented as a crenulation of the nanobeam. This reflector, in combination with the phononic mirror on the right side of the device, forms a Fabry-Pérot-like cavity for mechanical breathing modes. Examples of the mechanical breathing modes are shown in Fig. 1k, the frequencies and estimated vacuum optomechanical coupling rates of which are given in Table 1. Because the mechanical breathing modes extend along the beam, they can be directly actuated by electrodes that are spatially separated from the localized optical mode.

**Table 1:** Simulated and measured parameters for the dominant mechanical breathing modes.

	Simulated			Measured					
Mode	Ω_m_/2*π* [GHz]	*g*_0_/2*π* [kHz]	Γ_ex_/2*π* [mHz]	Ω_m_/2*π* [GHz]	*Q*_m_	*g*_0_/2*π* [kHz] (blue detuned)	*g*_0_/2*π* [kHz] (red detuned)	Γ_ex_/2*π* [mHz]	*ϕ*_m_/2*π* [rad]
A	3.173	173	6.20	3.280	1288	193	189	2.32	0
B	3.227	381	8.02	3.314	1180	285	281	3.80	*π*
C	3.249	506	25.2	3.328	1301	294	291	1.75	0

### Fabrication

**Fig. 2:**
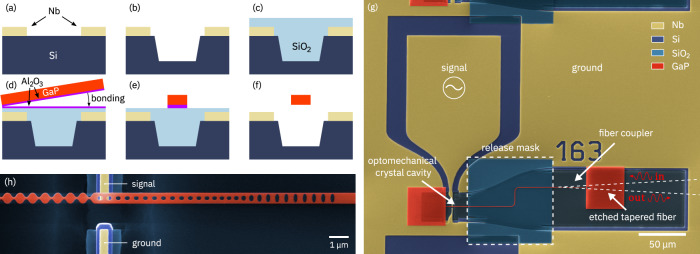
Device fabrication process. **a** Deposition and patterning of Nb electrodes. **b** Local recessing of the Si substrate. **c** Deposition and planarization of the sacrificial SiO_2_ layer. **d** Direct wafer bonding of the GaP device layer. **e** Patterning of the integrated GaP photonic circuit. **f** Release of the free-standing portion of the device. **g** False-color scanning electron microscope (SEM) image of the finished device with annotation of microwave and optical ports. **h** Close-up false-color SEM image of the optomechanical crystal cavity.

Device fabrication makes use of the processes described in our previous work on integrated GaP photonics^44,48^, with the important distinction that GaP is not bonded onto an oxidized silicon wafer but instead onto a prefabricated microwave circuit. The process flow is illustrated in Fig. 2. First, a 250 nm thick niobium film is deposited on an intrinsic silicon wafer by magnetron sputtering and patterned by chlorine-based dry etching into electrode structures. The vicinity of the photonic crystal cavity is then recessed 2 μm by dry etching of the silicon with a HBr/O_2_ mixture to minimize optical losses to the substrate. The wafer is then covered with a sacrificial SiO_2_ layer and the surface planarized for bonding of GaP. The thickness of the SiO_2_ layer determines the eventual gap (~300 nm) between the GaP beam and the underlying niobium electrode. The GaP source wafer comprises a 300 nm thick GaP device layer on top of an Al_0.1_Ga_0.9_P etch-stop layer on a 2-inch, [100]-oriented GaP substrate. Prior to direct bonding, a thin film (5 nm) of Al_2_O_3_ is deposited on both the GaP device layer and the target wafer. After removal of the substrate and etch-stop layer, the bonded wafer is diced into chips, and the GaP device layer is patterned by electron-beam lithography to form the photonic crystal cavity and the attached waveguide. The waveguide has a tapered end for adiabatic coupling to a fiber with a tapered tip formed by etching. The free-standing portion of the device is defined photolithographically and released by removal of the sacrificial SiO_2_ layer with buffered HF. Finally, the entire chip is coated with 8 nm of Al_2_O_3_ to protect the surface and prevent photooxidation during measurement. Further details, including a description of the adiabatic fiber coupling to the device, are given in the Supplementary Notes 1 and 3.

### Optomechanical characterization

**Fig. 3:**
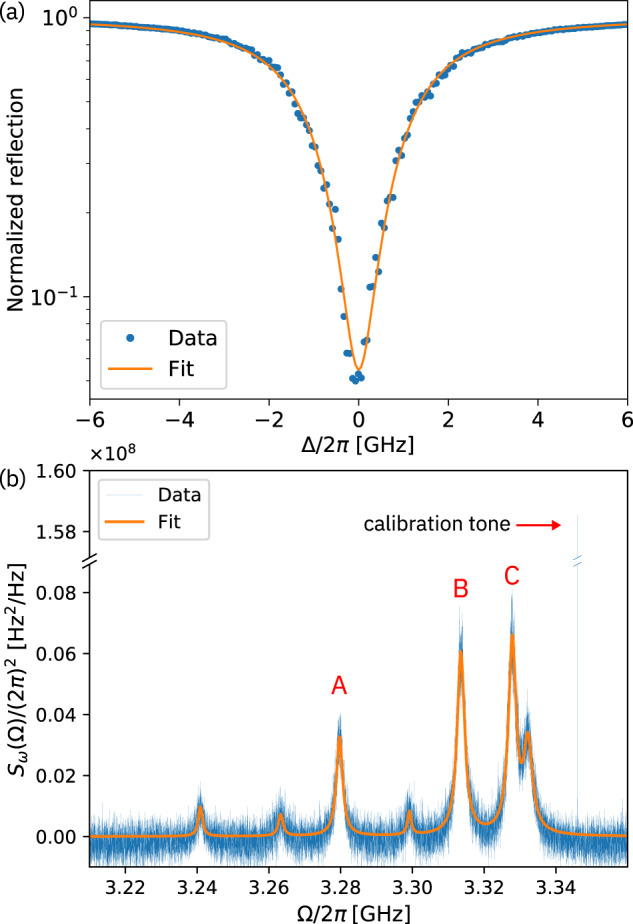
Optical and thermo-mechanical spectroscopy. **a** Fit (orange) of Eq. (1) to the optical reflection spectrum (blue) of the cavity resonance. **b** Thermo-mechanical spectrum (blue) plotted as symmetrized frequency noise spectral density *S*_*ω*_(Ω) with a fit to a sum of Lorentzian functions (orange). The data are calibrated with an added tone created by a phase modulator. The assignment to the modes shown in Fig. 1(k) is indicated by A, B, and C.

The optical cavity mode is first interrogated in reflection with a tunable diode laser at low power (~130 nW) to determine the resonance frequency *ω*_o_ and the loaded quality factor *Q*. For a Lorentzian resonance, reflection from the cavity as a function of detuning Δ = *ω*_L_ − *ω*_o_ with respect to the laser frequency *ω*_L_ is described by

1 $$| {{{{{{{\mathcal{R}}}}}}}}({{\Delta }}){| }^{2}={\left|\frac{({\kappa }_{0}-{\kappa }_{{{{{{{{\rm{ex}}}}}}}}})/2-i{{\Delta }}}{({\kappa }_{0}+{\kappa }_{{{{{{{{\rm{ex}}}}}}}}})/2-i{{\Delta }}}\right|}^{2},$$

where *κ*_0_ is the intrinsic cavity decay rate and *κ*_ex_ is the external coupling rate^36^. A fit of (1) to the resonance observed at *ω*_o_/2*π* = 196.5 THz (Fig. 3a) gives a loaded optical quality factor *Q* = *ω*_o_/*κ* = 6.73 × 10^4^. The coupling factor is ${\eta }_{{{{{{{{\rm{c,opt}}}}}}}}}=\frac{{\kappa }_{{{{{{{{\rm{ex}}}}}}}}}}{{\kappa }_{{{{{{{{\rm{ex}}}}}}}}}+{\kappa }_{0}}=0.383$, yielding an intrinsic quality factor *Q*_0_ = 1.09 × 10^5^. Similar values have been achieved with GaP optomechanical crystal cavities reported in other work^44,45^. In addition to surface roughness and fabrication imperfections, absorption in the bulk of the GaP or at the surface may be limiting the intrinsic quality factor. We also find an optical mode frequency that is 1.7% higher than expected from simulations, which we attribute to small deviations from the design geometry.

Thermally driven mechanical motion of the photonic crystal cavity modulates the cavity resonance frequency, which, for a detuned probe laser, produces amplitude fluctuations in the reflected light. We measure the resulting thermo-mechanical spectrum by direct detection with a fast photoreceiver. At frequencies above 500 MHz, the only modes with appreciable optomechanical coupling are observed in a narrow band between 3.21 and 3.35 GHz (Fig. 3b), in good agreement with the simulated mechanical breathing modes. As the total optical decay rate is measured to be *κ* = *κ*_0_ + *κ*_ex_ = 2*π* × 2.92 GHz, the system is narrowly in the resolved-sideband regime.

The vacuum optomechanical coupling rate *g*_0_ of individual modes was determined via noise calibration^49^. To that end, the thermo-mechanical spectrum is measured at low cooperativity (${{{{{{{\mathcal{C}}}}}}}}={\bar{n}}_{{{{{{{{\rm{cav}}}}}}}}}\frac{4{g}_{0}^{2}}{{{{\Gamma }}}_{{{{{{{{\rm{m}}}}}}}}}\kappa }\approx 6.9\times 1{0}^{-3}$, where Γ_m_ is the mechanical damping rate and ${\bar{n}}_{{{{{{{{\rm{cav}}}}}}}}}$ is the average number of photons in the cavity), so that effects due to dynamical backaction can be neglected. A reference signal generated by a phase modulator is added close to the mechanical modes to calibrate the spectrum. The modulation depth *ϕ*_0_ = *πV*_0_/*V*_*π*_ (where *V*_0_ is the amplitude of the radio-frequency voltage applied to the modulator and *V*_*π*_ is the half-wave voltage) at frequency ${{{\Omega }}}_{{{{{{{{\rm{mod}}}}}}}}}$ is determined from the first sideband ratio (see Supplementary Note 11). The vacuum optomechanical coupling rate is then calculated as^49^

2 $${g}_{0}\approx \frac{{\phi }_{0}}{2}{{{\Omega }}}_{{{{{{{{\rm{m}}}}}}}}}\sqrt{\frac{1}{\langle {n}_{{{{{{{{\rm{th}}}}}}}}}\rangle }\frac{{S}_{V}({{{\Omega }}}_{{{{{{{{\rm{m}}}}}}}}}){{{\Gamma }}}_{{{{{{{{\rm{m}}}}}}}}}/4}{{S}_{V}({{{\Omega }}}_{{{{{{{{\rm{mod}}}}}}}}}){f}_{{{{{{{{\rm{ENBW}}}}}}}}}}},$$

where $\langle {n}_{{{{{{{{\rm{th}}}}}}}}}\rangle \approx \frac{{k}_{B}T}{\hslash {{{\Omega }}}_{{{{{{{{\rm{m}}}}}}}}}}$ is the average thermal occupation of the mode, *S*_*V*_(Ω) is the symmetrized voltage noise spectral density produced by the photoreceiver, Γ_m_ is the mechanical damping rate, and *f*_ENBW_ is the effective noise bandwidth of the spectrum analyzer’s filter function. We fit the mechanical noise spectrum with a sum of Lorentzians, as each mode produces a voltage noise spectral density that is uncorrelated with the other modes.

The device exhibits appreciable optomechanical coupling (*g*_0_/2*π* > 100 kHz) for three modes in the frequency range ascribed to the breathing modes, which we tentatively assign to the modes depicted in Fig. 1k. The observed mechanical resonance frequencies and coupling rates are listed in Table 1. We attribute the higher frequency of the experimentally observed modes to the Al_2_O_3_ protective coating, which increases the stiffness of the nanobeam (see Supplementary Note 4). The noise calibration measurement was carried out at blue and red laser detuning; the coupling rates for blue detuning are slightly higher than for red detuning, consistent with a small contribution from residual dynamical backaction.

### Piezoelectric coupling

**Fig. 4:**
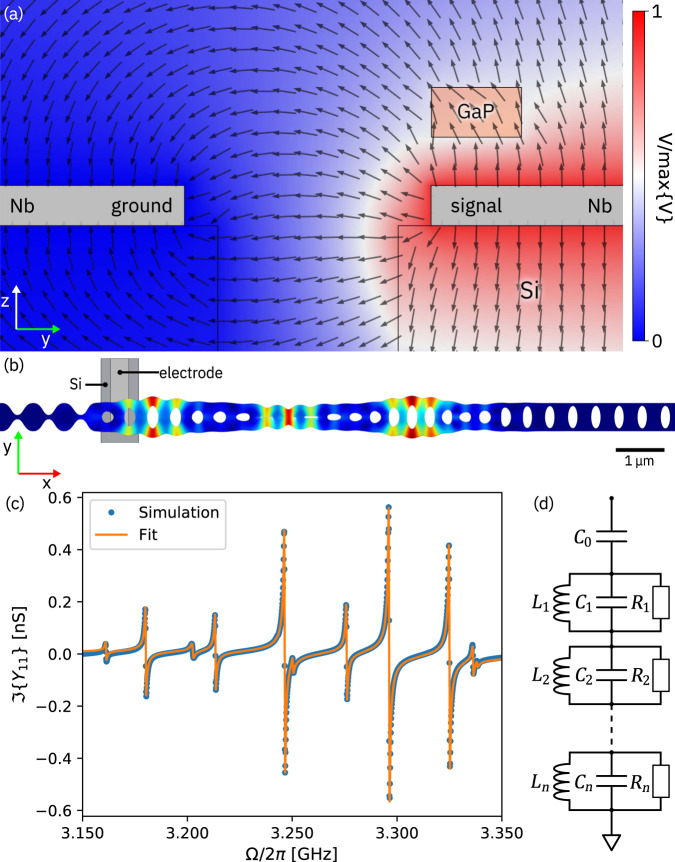
FEM simulation of piezoelectric coupling. **a** Cross-section of the electric potential between the coplanar microwave signal (red) and ground (blue) electrodes. The black arrows indicate the electric field direction. The orange box is the GaP beam. **b** FEM simulation of mechanical breathing mode C showing the position of the signal electrode. **c** Imaginary part of the simulated piezoelectric admittance *Y*_11_ (blue) and fit to an LC network (orange). **d** Circuit representation of the series network of parallel LC resonators used for the admittance fit.

We now turn the focus of the discussion to piezoelectrically mediated actuation of the optomechanical cavity and analyze the frequency dependence of the coupling with a multiphysics FEM simulation in which the signal voltage is applied to the electrode directly under the GaP beam and the other electrode is ground (Fig. 4). The zinc-blende crystal structure of GaP dictates that mechanical breathing modes are most effectively actuated with the axis of the optomechanical crystal cavity aligned with the [011] direction of the GaP crystal lattice and the electric field oriented along the [100] direction (*z*-axis in Fig. 4). As can be seen from Fig. 4a, the electric field for our coplanar electrode geometry has a component along the *z*-axis but is in general oriented at an angle. The coplanar electrode arrangement simplifies fabrication but is clearly not ideal. An optimized geometry is discussed below.

The admittance *Y*_11_ of the device as a function of frequency is calculated and fit to that of an equivalent network consisting of a series of parallel LC resonators^50^ (Fig. 4e). A small imaginary part is introduced to the elasticity tensor of GaP to broaden the spectral response of the individual modes and permit a coarser sampling of the spectrum. The resulting added loss is modeled as parallel resistors in the LC elements. The imaginary part of the admittance is shown in Fig. 4d along with the fit to the LC network, where the electrostatic contribution of the coupling capacitor *C*_0_ (which is determined by the geometry and dominates the admittance) has been subtracted for clarity. We find that the equivalent network provides an accurate representation of the electromechanical coupling.

With an equivalent circuit model in place, we can estimate the coupling of each mechanical mode to the microwave probe used experimentally by employing a semi-infinite transmission-line model. Specifically, the coupling capacitor *C*_0_ is assumed to be connected to a load impedance *Z*_0_ = 50 Ω, yielding for the *n*th mode an electromechanical coupling rate of

3 $${{{\Gamma }}}_{{{{{{{{\rm{ex}}}}}}}},n}\approx \frac{{Z}_{0}{C}_{0}^{2}{{{\Omega }}}_{{{{{{{{\rm{m}}}}}}}},n}^{2}}{{C}_{n}+{C}_{0}},$$

with ${{{\Omega }}}_{{{{{{{{\rm{m}}}}}}}},n}^{2}=1/{L}_{n}{C}_{n}$ (see Supplementary Note 7 for details). The values simulated for Γ_ex,*n*_ with a coupling capacitance of *C*_0_ = 0.42 fF are listed in Table 1.

### Microwave-to-optical transduction

**Fig. 5:**
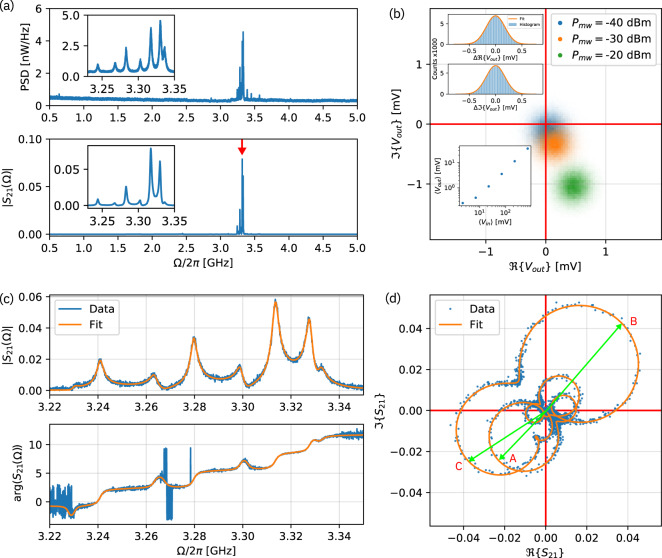
Microwave-to-optical transduction. **a** Thermo-mechanical noise power spectral density (PSD) (top) and microwave-to-optical transmission amplitude ∣*S*_21_∣ (bottom). **b** Quadrature components of the demodulated photoreceiver output voltage distribution *V*_out_ when the device is driven at 3.317 GHz (indicated by red arrow in the bottom panel of **a**) at three microwave powers. For each power level, 10^5^ points were collected at a bandwidth of 10 kHz. The insets show histograms of the real and imaginary part of the noise distribution at −20 dBm along with Gaussian fits (upper left) and the average output voltage 〈*V*_out_〉 versus the average microwave input voltage 〈*V*_in_〉 (lower left). **c** Amplitude and phase of the transduced signal versus drive frequency, where the contribution due to cable delay *τ* has been removed. **d** In-phase versus quadrature component of the transduced signal. Actuation at the measured eigenfrequencies of the dominant modes is indicated by green phasors, with the assignment to the modes shown in Fig. 1k indicated by A, B, and C.

We demonstrate microwave-to-optical transduction by driving the electrode below the device with a vector network analyzer (VNA) and measuring the optical signal from a detuned pump laser reflected from the cavity. The optomechanically induced modulation is observed by direct detection with a fast photoreceiver. The results are shown in Fig. 5. We evaluate the microwave-to-optical transduction by considering the system as a two-port network in which port 1 is the microwave probe and port 2 the photoreceiver. The scattering parameter *S*_21_(Ω) as a function of the VNA frequency Ω is then given by

4 $${S}_{21}({{\Omega }})=	 \,\frac{{{{\Gamma }}}_{{{{{{{{\rm{D}}}}}}}}}}{\sqrt{2{Z}_{0}\hslash {{\Omega }}}}{{\Theta }}({{\Omega }}){e}^{-i({{\Omega }}\tau +\theta )}\\ 	\cdot \mathop{\sum}\limits_{n}{g}_{0,n}\sqrt{{{{\Gamma }}}_{{{{{{{{\rm{ex}}}}}}}},n}}{\chi }_{{{{{{{{\rm{m}}}}}}}},n}({{\Omega }}){e}^{i{\phi }_{{{{{{{{\rm{m}}}}}}}},n}},$$

as derived in Supplementary Note 9. Here, Γ_D_ denotes a detection gain factor that is determined by the optical pump power, the photoreceiver’s quantum efficiency, as well as all electrical and optical loss and gain in the system. The subscript *n* denotes the parameters for the *n*th mechanical mode. The mechanical susceptibility is

5 $${\chi }_{{{{{{{{\rm{m}}}}}}}},n}({{\Omega }})=\frac{1}{\frac{{{{\Gamma }}}_{{{{{{{{\rm{m}}}}}}}},n}}{2}-i({{\Omega }}-{{{\Omega }}}_{{{{{{{{\rm{m}}}}}}}},n})}$$

with the mechanical damping rate Γ_m,*n*_ and eigenfrequency Ω_m,*n*_. The signal delay through the cables and optical fiber is described by a frequency dependent phase offset Ω*τ* + *θ*, where *τ* is the propagation time through the signal path and *θ* is a fixed arbitrary phase offset. Additionally, there is a mode-dependent phase offset *ϕ*_m,*n*_ ∈ {0, *π*}, corresponding to the relative phase difference between the mechanical displacement at the electromechanical and the optomechanical coupling positions. The optomechanically induced phase modulation is transduced into amplitude modulation detected by the photoreceiver according to the function

6 $${{\Theta }}({{\Omega }})=	\,i{\kappa }_{{{{{{{{\rm{ex}}}}}}}}}\big(\left(1-{\kappa }_{{{{{{{{\rm{ex}}}}}}}}}{\chi }_{{{{{{{{\rm{o}}}}}}}}}^{* }(0)\right){\chi }_{{{{{{{{\rm{o}}}}}}}}}(0){\chi }_{{{{{{{{\rm{o}}}}}}}}}({{\Omega }})\\ 	- \left(1-{\kappa }_{{{{{{{{\rm{ex}}}}}}}}}{\chi }_{{{{{{{{\rm{o}}}}}}}}}(0)\right){\chi }_{{{{{{{{\rm{o}}}}}}}}}^{* }(0){\chi }_{{{{{{{{\rm{o}}}}}}}}}^{* }(-{{\Omega }})\big),$$

where

7 $${\chi }_{{{{{{{{\rm{o}}}}}}}}}({{\Omega }})=\frac{1}{\frac{\kappa }{2}-i({{\Delta }}+{{\Omega }})}$$

is the optical susceptibility. The measured microwave-to-optical transmission amplitude ∣*S*_21_∣ is shown in Fig. 5a, along with the thermo-mechanical spectrum for comparison. The clear correspondence between the spectra indicates that the transduction is indeed mechanically mediated.

We verify the phase-coherence of the transduced signal by recording the distribution of the photoreceiver output voltage demodulated by the VNA at a fixed frequency of 3.317 GHz (where the highest transmission magnitude was observed), as displayed in Fig. 5b. The symmetric Gaussian distribution, which is dominated by thermo-mechanical noise, and the linear dependence on microwave input power of the displacement in-phase space at constant phase confirm that the transduction is linear and coherent.

The amplitude and phase of the transduction spectrum in the relevant range from 3.22 to 3.35 GHz are shown in Fig. 5c together with a fit to (4). The fit makes use of a separate calibration of the optical cavity response to determine Γ_D_, performed by phase-modulating the optical pump with a pre-determined modulation depth (see Supplementary Note 10). Using the previously measured vacuum optomechanical coupling rates, the electromechanical coupling rate Γ_ex_ is then determined from the microwave-to-optical transduction signal. The fit results for the three mechanical modes with the strongest transduction, which we assign to modes A, B, and C, are shown in Table 1. It is instructive to visualize the transduced signal in a phase space representation (Fig. 5d), where the harmonic response of the individual resonator modes follows a circular trajectory. In addition to a small rotation of each mode around the origin that is due to the optical cavity response, we observe a phase offset *ϕ*_m_ of each mode of either 0 or *π*. We understand this phenomenon to be a result of the shape of each breathing mode’s displacement field. When a mode is driven coherently, the mechanical displacement field in the portion of the GaP beam that overlaps with the electrode inherits its relative phase from the microwave drive. The optical mode, however, couples to a different region that might be in phase or out of phase by *π* with respect to the piezoelectrically coupled region (see Fig. 1k). Consequently, the optomechanically induced phase modulation of the optical field may also have an added phase offset of 0 (modes A and C) or *π* (mode B) with respect to the microwave drive. Note that, although the device possesses several other breathing modes, only the three modes A, B, and C provide a reasonable qualitative agreement between simulations and experiment with respect to mechanical frequency, optomechanical coupling rate, and mode-dependent phase offset, justifying the assignment.

### Outlook for coupling to a transmon qubit

The total transduction efficiency of the system is given by^30^

8 $$\eta ={\eta }_{{{{{{{{\rm{c}}}}}}}},{{{{{{{\rm{mw}}}}}}}}}{\eta }_{{{{{{{{\rm{c}}}}}}}},{{{{{{{\rm{opt}}}}}}}}}\frac{4{{{{{{{\mathcal{C}}}}}}}}}{{(1+{{{{{{{\mathcal{C}}}}}}}})}^{2}},$$

where ${{{{{{{\mathcal{C}}}}}}}}={\bar{n}}_{{{{{{{{\rm{cav}}}}}}}}}\frac{4{g}_{0}^{2}}{\kappa {{{\Gamma }}}_{{{{{{{{\rm{m}}}}}}}}}}$ is the optomechanical cooperativity, and ${\eta }_{{{{{{{{\rm{c}}}}}}}},{{{{{{{\rm{mw}}}}}}}}}=\frac{{{{\Gamma }}}_{{{{{{{{\rm{ex}}}}}}}}}}{{{{\Gamma }}}_{{{{{{{{\rm{m}}}}}}}}}}$ and ${\eta }_{{{{{{{{\rm{c}}}}}}}},{{{{{{{\rm{opt}}}}}}}}}=\frac{{\kappa }_{{{{{{{{\rm{ex}}}}}}}}}}{\kappa }$ are the external microwave and optical coupling factors to the system, respectively. With ${{{{{{{\mathcal{C}}}}}}}}=6.9\times 1{0}^{-3}$ at an optical pump power of 130 nW, we find a maximum transduction efficiency for the device as measured of *η* = 1.4 × 10^−11^. The efficiency can be increased by raising the optical power to achieve the optimal value of ${{{{{{{\mathcal{C}}}}}}}}=1$, but the efficiency would still be quite low because of the extremely low microwave external coupling factor *η*_c,mw_ = 1.35 × 10^−9^, which is a consequence of coupling to a highly impedance-mismatched transmission line instead of a resonant microwave cavity. As such, this efficiency value is misleading.

**Fig. 6:**
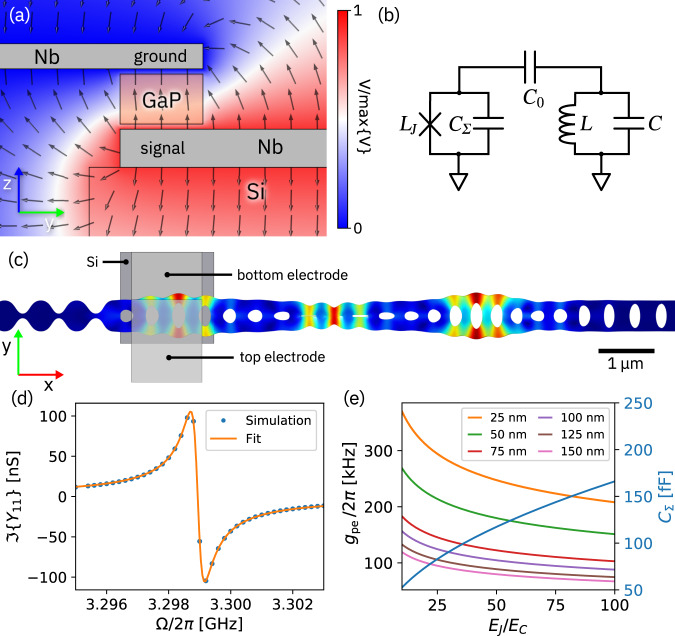
Theoretical analysis of coupling to a transmon qubit. **a** Cross-section of a FEM simulation of the electric potential between the microwave signal (red) and ground (blue) electrodes for the optimized geometry. The black arrows indicate the electric field direction. The orange box is the GaP beam. **b** Circuit representation of a transmon qubit coupled to a parallel LC resonator. **c** FEM simulation of a mechanical breathing mode showing the electrode geometry. **d** Imaginary part of the simulated piezoelectric admittance *Y*_11_ (blue) and fit to an LC resonator (orange). **e** Calculated qubit shunt capacitance *C*_Σ_ and coupling rates to a transmon qubit as function of *E*_*J*_/*E*_*C*_ for various gap sizes between the electrodes and the photonic crystal cavity.

Instead, we consider the more meaningful situation of coupling to a superconducting transmon qubit and estimate the expected electromechanical coupling rate. We restrict the discussion here to an optimized configuration with one electrode below and the other above the photonic crystal cavity with equal gaps to the electrodes (Fig. 6a). The capacitive qubit-resonator coupling scheme is depicted in Fig. 6b, with the circuit parameters *C*_0_, *L*, and *C* extracted from an admittance fit similar to that described above. In this case, we simulate only the admittance of mechanical mode C, as it exhibits the highest simulated electromechanical coupling in the coplanar geometry. The bilinear coupling between the qubit and the resonator is given by

9 $${g}_{{{{{{{{\rm{pe}}}}}}}}}=\frac{1}{2}{C}_{0}\sqrt{\frac{{{{\Omega }}}_{{{{{{{{\rm{ge}}}}}}}}}{{{\Omega }}}_{{{{{{{{\rm{m}}}}}}}}}}{{C}_{{{\Sigma }}}C}},$$

where *C*_Σ_ is the qubit shunt capacitance, and the first qubit transition frequency is Ω_ge_ (see Supplementary Note 8 for details). For optimal coupling, we assume that the qubit is tuned into resonance with the mechanical oscillator, i.e., Ω_ge_ = Ω_m_. The estimated coupling rate is shown in Fig. 6e for various gap sizes between the electrodes and the optomechanical crystal cavity as a function of the ratio of Josephson energy *E*_*J*_ to charging energy of the qubit *E*_*C*_. For the qubit to exhibit low charge dispersion, a ratio *E*_*J*_/*E*_*C*_ ≫ 1 is required^51^. For a gap to the electrodes of 50 nm, which can be realistically fabricated, we calculate a coupling rate of *g*_pe_/2*π* = 204 kHz for *E*_*J*_/*E*_*C*_ = 30, well within the transmon regime, with *C*_Σ_ = 91 fF. Previously, mechanical quality factors of *Q*_m_ ≈ 2 × 10^5^ have been reported for similar GaP optomechanical crystal cavities at the cryogenic temperature required for single-phonon manipulation^45^, which corresponds at Ω_m_/2*π* = 3 GHz to a mechanical damping rate of Γ_m_/2*π* = 15 kHz. We therefore estimate that the system presented here would be deep enough in the strong coupling regime to permit a faithful swap of the qubit and mechanical resonator states, if the qubit lifetime *T*_1_ ≳ 10 μs. Given the small contribution of the piezoelectric portion (*C*_0_ ≈ 0.79 fF) to the total capacitance of such a system, we expect this *T*_1_ to be achievable with current technology.

## Discussion

In summary, we have presented a platform for microwave-to-optical conversion based on single-crystal GaP optomechanical devices integrated directly on prefabricated niobium-on-silicon electrode structures. The extended mechanical breathing modes of our optomechanical crystal cavities were designed for both high optomechanical coupling rates (up to *g*_0_/2*π* = 300 kHz) and piezoelectric actuation by the microwave electrodes. Despite the asymmetric cavity structure and the close proximity of the metal electrodes, high optical quality factors (*Q*_0_ ~ 10^5^) were obtained, placing the system in the resolved-sideband regime (Ω_m_/*κ* ~ 1.1). Coherent microwave-to-optical transduction in a transmission-line coupled device was demonstrated. The electromechanical coupling rates observed experimentally were substantially smaller than expected from simulations. The discrepancy could be fabrication-related or may be due to insufficient knowledge of the piezoelectric properties of GaP. In future experiments, we aim to integrate the cavity design presented here with superconducting transmon qubits, for which our simulations indicate that strong coupling can be achieved (*g*_pe_/2*π* ≈ 200 kHz). Due to the small participation ratio of the device in the overall qubit energy, we expect long qubit lifetimes.

## Supplementary information

Supplementary Information

## Data Availability

Data supporting the plots within this paper and other findings of this study are available through Zenodo at 10.5281/zenodo.6419115. Further information is available from the corresponding author upon reasonable request.

## Code availability

Numerical codes used in this study are available from the corresponding author upon reasonable request.
