# Supplementary material for: Microwave-to-optical conversion with a gallium phosphide photonic crystal cavity
Source: Nat Commun. 2022 Apr 19;13:2065. doi: 10.1038/s41467-022-28670-5 (PMC9019098; doi:10.1038/s41467-022-28670-5)
Supplement: Supplementary file 1 — Supplementary Information [file 41467_2022_28670_MOESM1_ESM.pdf]

Supplementary Information for "Microwave-to-optical conversion with a gallium phosphide photonic crystal cavity"

Simon Hönl,[1] Youri Popoff,[1,2] Daniele Caimi,[1] Alberto Beccari,[3] Tobias J. Kippenberg,[3] and Paul Seidler[1,*]

[1] *IBM Quantum, IBM Research Europe, Zurich, Säumerstrasse 4, CH-8803 Rüschlikon, Switzerland*
[2] *Integrated Systems Laboratory, Swiss Federal Institute of Technology Zurich (ETH Zürich), CH-8092 Zürich, Switzerland*
[3] *Institute of Physics, Swiss Federal Institute of Technology Lausanne (EPFL), CH-1015 Lausanne, Switzerland*

Information supplementary to the main text is presented here, including details of device fabrication, characterization methods, and derivation of the models applied.

SUPPLEMENTARY NOTE 1 – DEVICE FABRICATION

Here, we provide additional details on the fabrication process shown in Fig. 2 of the main text. We also refer the reader to [1–3].

Microwave electrode structures are fabricated by magnetron sputtering of a 250 nm-thick niobium film on a 4-inch intrinsic silicon wafer. The niobium film is patterned by inductively coupled-plasma reactive ion etching (ICP-RIE) with a Cl/Ar mixture using a SiO_2 hard mask. The silicon substrate is recessed 2 µm locally in the regions where the photonic crystal cavities will be placed using ICP-RIE with a HBr/O_2 gas mixture. The wafer is then covered with a sacrificial layer of SiO_2 deposited by plasma-enhanced chemical vapor deposition from a mixture of SiH_4 and N_2O. The surface is planarized for bonding of GaP by first etching back the higher regions by ICP-RIE using a mixture of C_4F_8 and O_2 followed by chemical mechanical polishing (CMP). An optical micrograph and an atomic force microscope (AFM) scan of the planarized surface are shown in Supplementary Fig. 1. Note that the topography introduced by the substrate recess is completely removed. After several repolishing steps, we obtain a surface roughness of < 5 Å over a $5\,\mu m \times 5\,\mu m$ area, sufficient for direct wafer bonding.

The 300 nm-thick GaP layer to be bonded to the above wafer is grown epitaxially by metal-organic chemical vapor deposition on top of an $Al_{0.1}Ga_{0.9}P$ etch stop layer on a 2-inch, [100]-oriented GaP wafer. After bonding, the majority of the GaP growth substrate is removed by non-selective wet etching in a solution of potassium ferricyanide ($K_3Fe(CN)_6$) and KOH (Transene Gallium Phosphide Etchant); the remaining 50–100 µm of the substrate wafer are eliminated by selective ICP-RIE with a mixture of $SiCl_4$ and SF_6, as described in our earlier work [2]. The $Al_{0.1}Ga_{0.9}P$ etch stop layer is subsequently removed with concentrated HCl.

After dicing into chips, the GaP device layer is patterned to form the photonic crystal cavity and the attached waveguide by electron-beam lithography using 6 % hydrogen silsesquioxane (HSQ) as resist (90 nm nominal thickness). The pattern is transferred by ICP-RIE using a $Cl_2/BCl_3/H_2/CH_4$ gas mixture. To promote adhesion of the HSQ, the surface of the GaP is coated by atomic layer deposition (ALD) with 3 nm of SiO_2 prior to spin-coating. The HSQ mask is removed with a dip in buffered hydrofluoric acid. The freestanding portion of the device is then defined photolithographically and released by removal of the sacrificial SiO_2 layer with buffered HF. Finally, the entire chip is coated with 8 nm of Al_2O_3 by ALD to protect the surface and mitigate photooxidation during measurement.

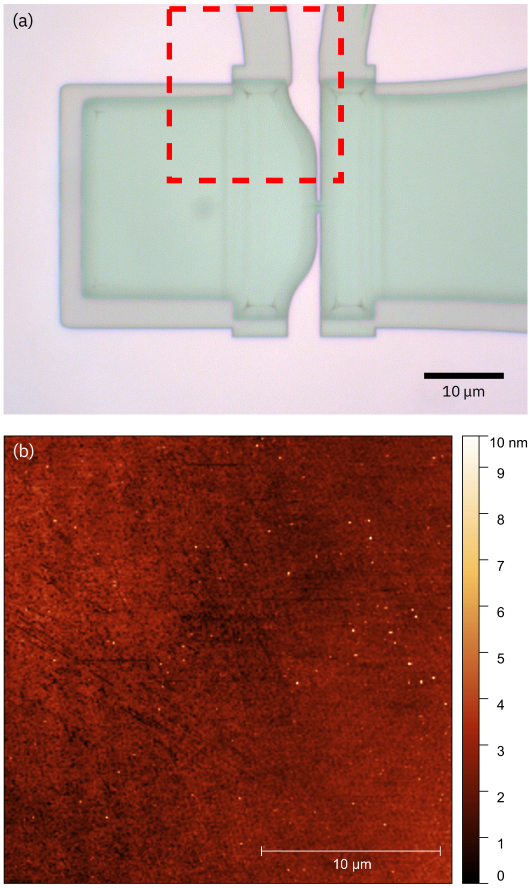

SUPPLEMENTARY FIG. 1. **Surface after CMP**. (a) Optical micrograph of a portion of the device structure before wafer bonding, where the red, dashed box indicates the location of the AFM scan shown in (b).

SUPPLEMENTARY NOTE 2 – INTEGRATION OF A TOP ELECTRODE

Piezoelectric coupling can be improved by selecting an electrode geometry that provides a better overlap between the electric field produced by the electrodes and the breathing-mode displacement field of the photonic crystal cavity. To that end, and as discussed in the main text, an electrode may be positioned above the GaP beam. Following the step of patterning the GaP (Fig. 2e in the main text), a SiO_2 cladding is deposited on top of the photonic circuit and planarized by CMP. A via is then etched through the cladding down to the buried metal layer and another niobium film is deposited by magnetron sputtering. The top electrode is etched with the same process as used for the bottom niobium structures. The process steps are outlined in Supplementary Fig. 2(a-d). The device is then released as described above. Scanning electron microscope (SEM) images of a cross-section made by focused-ion-beam milling prior to release and of the final device are shown in Supplementary Fig. 2(e-g).

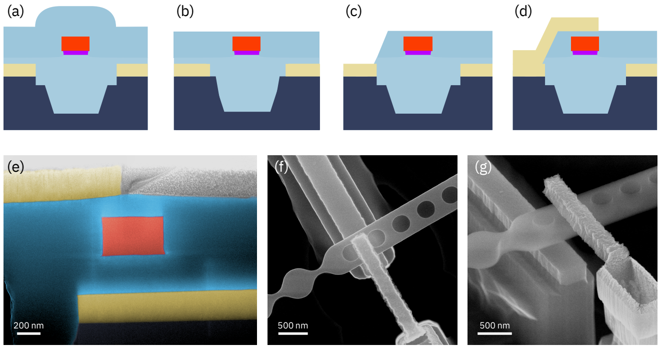

SUPPLEMENTARY FIG. 2. **Fabrication process for devices with top electrode**. (a) Deposition of SiO_2 cladding. (b) Planarization by CMP. (c) Etching of via. (d) Deposition and patterning of top niobium electrode. (e) False-color SEM image of a cross-section through the optomechanical crystal cavity at the location of the electrodes made by focused-ion-beam milling. (f, g) SEM images of the optomechanical crystal cavity in the region of the electrodes.

SUPPLEMENTARY NOTE 3 – ADIABATIC FIBER COUPLING

The devices in the main text are coupled to the experimental setup using an adiabatic fiber interface. We followed the implementation by Burek et al. [4] for the fabrication of the tapered fibers and the design of the waveguide coupler. Optical fibers (Corning® SMF-28® Ultra) are etched in concentrated hydrofluoric acid (47 %) covered with a layer of xylene. The xylene assists the formation of a meniscus around the optical fiber. The fibers are mounted on a modified 3D printer (Wanhao Duplicator i3 mini), which is programmed to slowly retract the fiber from the etching bath. In this way, tapers could be reproducibly fabricated with a specific angle (here 2.85°) and with an apex radius < 50 nm. A typical taper is shown in Supplementary Fig. 3(b). Close to the fiber tip, the optical mode expands around the etched silica core as the effective refractive index drops. Finite-element-method (FEM) simulations of cross-sections of the optical mode along the taper for a wavelength $\lambda_{\mathrm{vac}} = 1550$ nm are shown in Supplementary Fig. 3(a). The effective index of the fiber taper is shown in Supplementary Fig. 3(c) as a dashed green line for a tip diameter tapering from 8 µm at $x/L_{\mathrm{coupler}} = 0$ to 100 nm at $x/L_{\mathrm{coupler}} = 1$. The strong evanescent field enables coupling to a similarly tapered GaP waveguide. The dashed orange line in Supplementary Fig. 3(c) indicates the effective refractive index for an air-cladded, 300 nm-thick GaP waveguide tapering from a width of 150 nm at $x/L_{\mathrm{coupler}} = 0$ to 350 nm at $x/L_{\mathrm{coupler}} = 1$. The solid blue line in Supplementary Fig. 3(c) indicates the effective refractive index of the hybridized optical mode that forms when the fiber and waveguide are brought into contact. The transition from confinement in the optical fiber to confinement in the waveguide is evident from the cross-sections of the electric field distribution displayed in Supplementary Fig. 3(a). An adiabatic transition can be achieved if $L_{\mathrm{coupler}} \gg \lambda_{\mathrm{vac}}$. We chose $L_{\mathrm{coupler}} = 50$ µm and verified a coupling efficiency of 70 % with a finite-difference time domain (FDTD) simulation (see Supplementary Fig. 3(d), where the color scale is adjusted to highlight the residual radiation loss of the coupler). Supplementary Fig. 4 shows the actual coupling efficiency between an etched fiber taper and a GaP waveguide determined by measuring the light reflected from the photonic crystal cavity off resonance. We attribute the excess measured loss to imperfections in the etched taper and additional loss in the GaP waveguide.

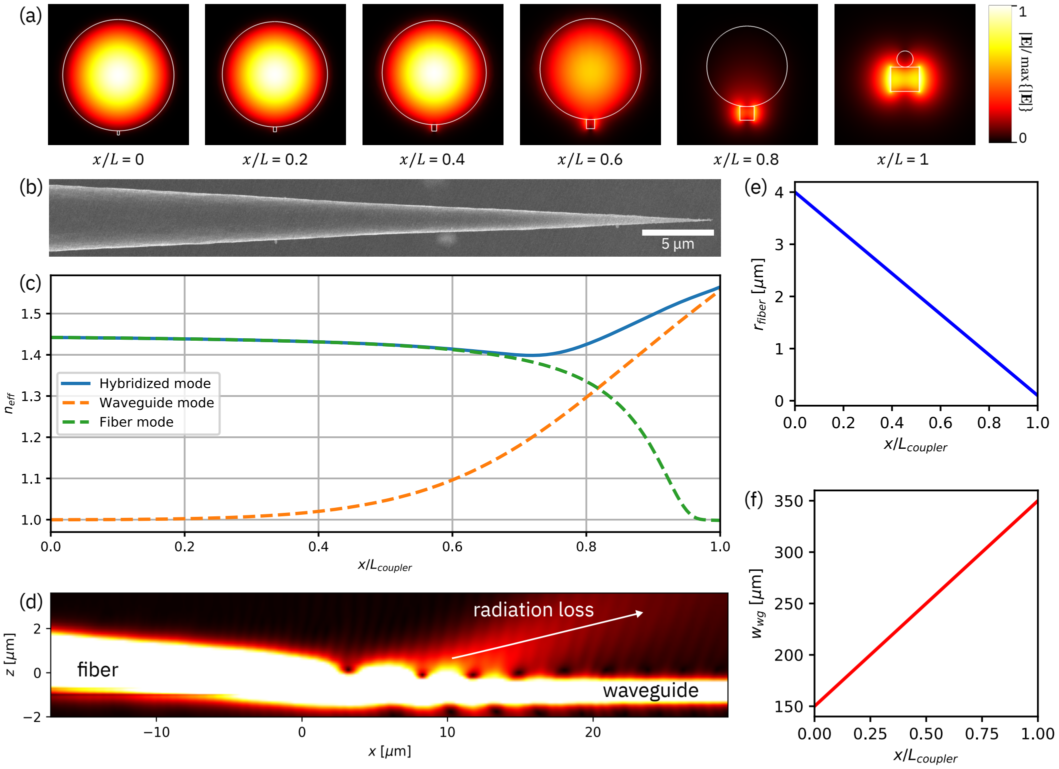

SUPPLEMENTARY FIG. 3. **Adiabatic fiber coupler.** (a) FEM simulations of cross-sections of the optical mode along the taper for a wavelength $\lambda_{\mathrm{vac}} = 1550\,\mathrm{nm}$. The color scale indicates the magnitude of the electric field $|\boldsymbol{E}|$. (b) SEM image of etched fiber tip. (c) Effective index of optical mode along the length L_{coupler} of the fiber coupler. (d) FDTD simulation of transmission from the fiber to the waveguide. (e,f) Fiber radius and waveguide width, respectively, as a function of the coordinate along the coupler.

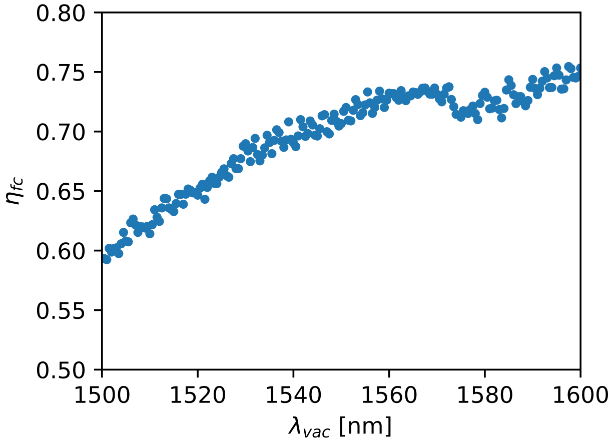

SUPPLEMENTARY FIG. 4. **Measured fiber coupling efficiency**. Coupling efficiency between fiber and waveguide η_{fc} measured by reflection from the photonic crystal cavity.

SUPPLEMENTARY NOTE 4 – IMPACT OF Al_2O_3 COATING ON MECHANICAL FREQUENCY

As mentioned in the main text, we attribute the increase in measured mechanical frequencies with respect to the simulated values at least partially to the protective Al_2O_3 coating added to the devices at the end of fabrication. Evidence for this conclusion is provided by the thermo-mechanical spectra before and after atomic-layer deposition of Al_2O_3 (Supplementary Fig. 5). We observe a frequency shift of $\sim 150\,\mathrm{MHz}$ for all mechanical modes, indicating an increased stiffness of the photonic crystal cavity that more than compensates for the added mass.

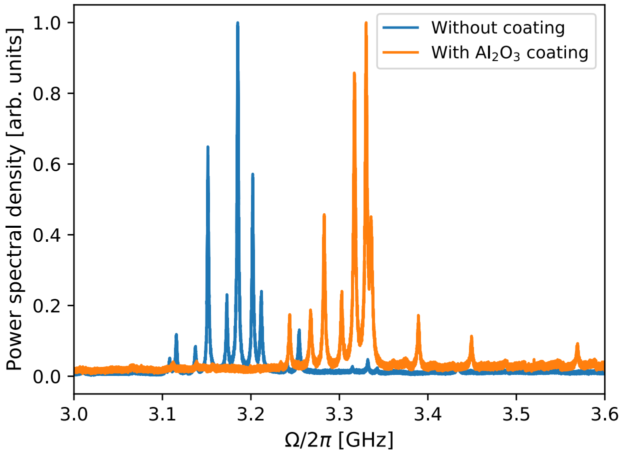

SUPPLEMENTARY FIG. 5. Normalized thermo-mechanical spectrum before (blue) and after (orange) deposition of Al_2O_3 as protective coating.

SUPPLEMENTARY NOTE 5 – EXPERIMENTAL SETUP

The optomechanical device is characterized with the setup shown in Supplementary Fig. 6(a). The optical pump is produced by an external-cavity diode laser (ECDL) and attenuated using a variable optical attenuator (VOA). Optical sidebands are added to the carrier using a lithium niobate electro-optic phase modulator (PM) driven by the RF source of a vector network analyzer (VNA). The device input power is determined at a 50 % tap of a fiber-optic beam splitter before sending the light to the device under test (DUT) through a fiber-optic circulator. Light is coupled to the integrated photonic circuit with an etched tapered fiber with a typical single-pass insertion loss of 1.5 dB. A fiber-optic beam splitter taps 10 % of the reflected light to measure the optical power for spectroscopy of the cavity mode and determination of the insertion loss. The remaining signal is amplified with an erbium-doped fiber amplifier (EDFA), and the amplified spontaneous emission background is filtered using a tunable optical bandpass filter (BP) with 0.8 nm bandwidth. A fiber-optic beam splitter is used to send 90 % of the filtered signal to a fast photoreceiver (RX), while 10 % is divided equally with another fiber-optic beam splitter between an optical power meter and an optical spectrum analyzer (OSA) used to adjust the bandpass filter. The output of the fast photoreceiver is sent either to the VNA or to an electrical spectrum analyzer (ESA).

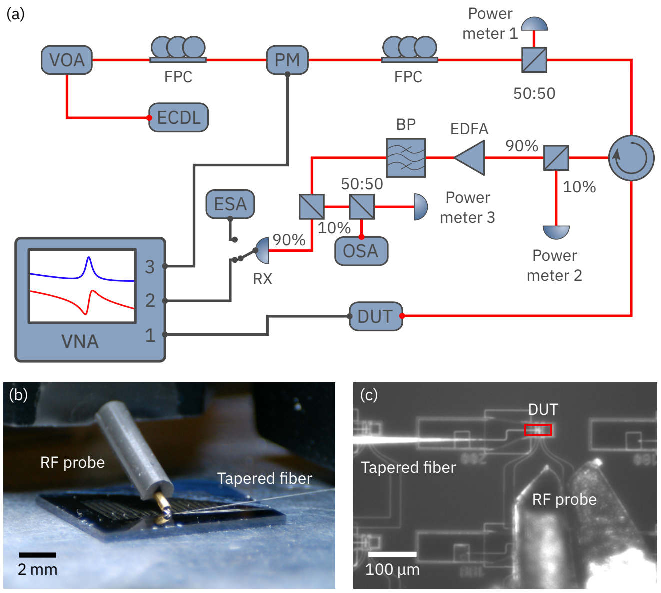

SUPPLEMENTARY FIG. 6. **Microwave-to-optical characterization apparatus**. (a) Schematic of apparatus. ECDL, external-cavity diode laser; VOA, variable optical attenuator; PM, phase modulator; FPC, fiber polarization controller; EDFA, erbium-doped fiber amplifier; BP, tunable bandpass filter; OSA, optical spectrum analyzer; ESA, electrical spectrum analyzer; DUT, device under test; VNA, vector network analyzer. (b) RF probe and tapered fiber aligned to sample. (c) Top view of sample in measurement apparatus.

SUPPLEMENTARY NOTE 6 – EQUIVALENT CIRCUIT FOR THE PIEZOELECTRIC RESPONSE

We describe the piezoelectric admittance of the device presented in the main text in terms of a series network of parallel RLC resonators as shown in Supplementary Fig. 7, each RLC resonator representing the piezoelectric response of a mechanical mode. The admittance at the input node (left side in Supplementary Fig. 7) can be expressed in terms of the network impedance $Z(\Omega)$ as $Y(\Omega) = Z^{-1}(\Omega)$, where

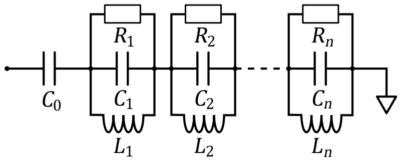

SUPPLEMENTARY FIG. 7. **Equivalent circuit for the piezoelectric response**. Series network of parallel RLC resonators coupled to an input node via a coupling capacitor C_0. Each mechanical mode is represented by a parallel RLC element with capacitance C_i, inductance L_i, and, to account for spectral broadening by mechanical loss, resistance R_i.

$$Z(\Omega) = Z_{\rm c}(\Omega) + \sum_{i=k}^{n} Z_k(\Omega). \tag{1}$$

Here, $Z_{\rm c}(\Omega) = 1/i\Omega C_0$ is the impedance of the input capacitor C_0, and $Z_k(\Omega) = Y_k^{-1}(\Omega)$ is the impedance of the k-th RLC node. The admittance $Y_k(\Omega)$ is given by

$$Y_k(\Omega) = \frac{1}{R_k} + \frac{1}{i\Omega L_k} + i\Omega C_k. \tag{2}$$

SUPPLEMENTARY NOTE 7 – ELECTROMECHANICAL COUPLING RATE TO A TRANSMISSION LINE

If the mechanical resonances are well separated, the electromechanical coupling rate $\Gamma_{\rm ex}$ to a transmission line for a given resonance may be estimated by considering only the RLC element of the network corresponding to that resonance. Moreover, the coupling rate is independent of R_i. We therefore analyze the case of a single, lossless ($R_i \to \infty$), parallel LC resonator coupled to a semi-infinite transmission line [5] via the coupling capacitor C_0. The environmental impedance acting on the LC resonator is given by

$$Z_{\rm env} = Z_0 + \frac{1}{i\Omega C_0} = \frac{1 + i\Omega C_0 Z_0}{i\Omega C_0}, \tag{3}$$

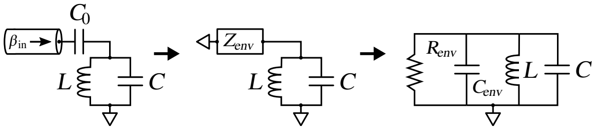

SUPPLEMENTARY FIG. 8. **Coupling to a semi-infinite transmission line**. (left) LC element coupled to a microwave input field β_{in} via a semi-infinite transmission line. The input is modelled as a $50\,\Omega$ load. (middle) Equivalent circuit, in which the environmental impedance Z_{env} determines the coupling rate of the LC resonator to the transmission line. (right) Equivalent circuit in which the environmental impedance is treated as a resistance and capacitance in parallel.

where the transmission line is considered to be an ideal resistor with $Z_0 = 50\,\Omega$. The environmental admittance parallel to the LC resonator is then

$$Y_{\text{env}} = Z_{\text{env}}^{-1} = \frac{i\Omega C_0 + \Omega^2 C_0^2 Z_0}{1 + \Omega^2 C_0^2 Z_0^2}. \quad (4)$$

We compare this expression for Y_{env} to that of an equivalent circuit consisting of a resistor and a capacitor in parallel (Supplementary Fig. 8), namely

$$Y_{\text{env}} = \frac{1}{R_{\text{env}}} + i\Omega C_{\text{env}} \quad (5)$$

The imaginary part of the environmental admittance,

$$\Im\{Y_{\text{env}}\} = \frac{\Omega C_0}{1 + \Omega^2 C_0^2 Z_0^2}, \quad (6)$$

simplifies for weak coupling ($C_0 Z_0 \ll CZ$ with $Z = \sqrt{L/C}$, i.e., the impedence of the LC resonator) to

$$\Im\{Y_{\text{env}}\} = \Omega C_{\text{env}} \approx \Omega C_0, \quad (7)$$

which corresponds to a capacitance C_0 in parallel with the capacitance C of the LC resonator, yielding $C_{\text{res}} = C + C_0$.

The real part of the environmental admittance determines the dissipation by the transmission line and, again for weak coupling, is given by

$$\Re\{Y_{\text{env}}\} = \frac{\Omega^2 C_0^2 Z_0}{1 + \Omega^2 C_0^2 Z_0^2} \approx \Omega^2 C_0^2 Z_0, \quad (8)$$

which corresponds to the conductance of a parallel resistor with resistance

$$R_{\text{env}} = \frac{1}{\Omega^2 C_0^2 Z_0}. \quad (9)$$

The decay rate of the LC resonator through the transmission line for the equivalent circuit is then [6]

$$\Gamma_{\text{ex}} = \frac{1}{R_{\text{env}} C_{\text{res}}} = \frac{\Omega^2 C_0^2 Z_0}{C + C_0}. \quad (10)$$

SUPPLEMENTARY NOTE 8 – PIEZOELECTRIC COUPLING RATE TO A TRANSMON QUBIT

We consider again a single, lossless LC element of the equivalent circuit depicted in Supplementary Fig. 7, except that now it is connected to a transmon qubit with Josephson energy $E_J = \varphi_0^2/L_J$, where φ_0 is the reduced flux quantum, and L_J is the effective inductance of the Josephson junction [5]. The Josephson junction is shunted with a capacitor C_Σ.

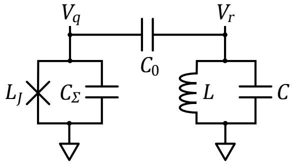

SUPPLEMENTARY FIG. 9. **Transmon qubit coupled to linear parallel LC resonator**. LC equivalent element with capacitance C and inductance L coupled via a capacitor with capacitance C_0 to a superconducting transmon qubit with Josephson inductance L_J and shunt capacitance C_Σ.

By considering the voltages $V_q = Q_\Sigma/C_\Sigma$ and $V_r = Q/C$ as depicted in Supplementary Fig. 9, we may calculate the charge Q_0 on the coupling capacitor C_0 via

$$V_q - V_r = \frac{Q_0}{C_0} = \frac{Q_\Sigma}{C_\Sigma} - \frac{Q}{C}, \quad (11)$$

where Q_Σ and Q are the charges on the transmon shunt capacitor and the capacitor of the LC resonator, respectively. The overall charging energy of the system is then

$$\begin{aligned} E_{C,\text{total}} =& \frac{Q_\Sigma^2}{2C_\Sigma} + \frac{Q^2}{2C} + \frac{C_0}{2}\left(\frac{Q_\Sigma}{C_\Sigma} - \frac{Q}{C}\right)^2 \\ =& \frac{Q_\Sigma^2}{2C_\Sigma}\left(1 + \frac{C_0}{C_\Sigma}\right) + \frac{Q^2}{2C}\left(1 + \frac{C_0}{C}\right) \\ &- C_0\left(\frac{Q_\Sigma Q}{C_\Sigma C}\right) \end{aligned} \quad (12)$$

In order to find the interaction Hamiltonian, we replace Q and Q_Σ with appropriate charge operators in second quantization

$$\begin{aligned} Q_\Sigma &\mapsto \sqrt{\frac{\hbar\Omega_{\text{ge}} C_\Sigma}{2}}\, i(\hat{c} - \hat{c}^\dagger) \\ Q &\mapsto \sqrt{\frac{\hbar\Omega_{\text{m}} C}{2}}\, i(\hat{b} - \hat{b}^\dagger), \end{aligned} \quad (13)$$

where Ω_{ge} is the qubit's first transition frequency, Ω_{m} is the mechanical resonator's frequency, and $\hat{c}$ and $\hat{b}$ are the annihilation operators of the qubit and the mechanical resonator modes, respectively. We obtain

$$\hat{Q}_\Sigma \hat{Q} = -\frac{\hbar}{2}\sqrt{\Omega_{\text{ge}}\Omega_{\text{m}} C_\Sigma C}\left(\hat{c}^\dagger\hat{b}^\dagger - \hat{c}^\dagger\hat{b} - \hat{c}\hat{b}^\dagger + \hat{c}\hat{b}\right), \quad (14)$$

and may thus rewrite the interaction term in Eq. 12 as

$$-C_0\left(\frac{\hat{Q}_\Sigma\hat{Q}}{C_\Sigma C}\right) = -\hbar g_{\mathrm{pe}}\left(\hat{c}^\dagger\hat{b}+\hat{c}\hat{b}^\dagger\right)+\hbar g_{\mathrm{pe}}\left(\hat{c}^\dagger\hat{b}^\dagger+\hat{c}\hat{b}\right) \equiv \hat{H}_{\mathrm{int}} \tag{15}$$

with the coupling rate

$$g_{\mathrm{pe}} = \frac{1}{2}C_0\sqrt{\frac{\Omega_{\mathrm{ge}}\Omega_{\mathrm{m}}}{C_\Sigma C}}. \tag{16}$$

In the case of resonant ($\Omega_{\mathrm{ge}} = \Omega_{\mathrm{m}}$) and sufficiently weak ($g_{\mathrm{pe}} \ll \Omega_{\mathrm{m}}$) coupling, we may use the rotating-wave approximation such that

$$\hat{H}_{\mathrm{int}} = -\hbar g_{\mathrm{pe}}\left(\hat{c}^\dagger\hat{b}+\hat{c}\hat{b}^\dagger\right). \tag{17}$$

We consider the transmon limit where $E_J/E_C \gg 1$. To find an appropriate shunt capacitance C_Σ for a given value of E_J/E_C, we use

$$\frac{E_J}{E_C} = \frac{\varphi_0^2}{L_J}\frac{2C_\Sigma}{e^2}, \tag{18}$$

which gives

$$C_\Sigma = \frac{1}{2}\frac{E_J}{E_C}\frac{L_J e^2}{\varphi_0^2}, \tag{19}$$

where e is the elementary charge. For a qubit frequency resonant with the mechanical mode, i.e.,

$$\Omega_{\mathrm{ge}}^2 = \frac{1}{L_J C_\Sigma} = \Omega_{\mathrm{m}}^2, \tag{20}$$

we find

$$C_\Sigma = \frac{e}{\Omega_{\mathrm{m}}\varphi_0}\sqrt{\frac{E_J}{2E_C}}. \tag{21}$$

SUPPLEMENTARY NOTE 9 – COHERENT MICROWAVE-TO-OPTICAL TRANSDUCTION IN THE LOW-COOPERATIVITY LIMIT

In the main text we present a linear response model that describes the scattering parameter S_{21} between the microwave probe connected to the device and the output of the photoreceiver. We may use input-output theory [5] to describe the transmitted signal. For simplicity, we operate in a regime where dynamical backaction is negligible, as verified by the noise calibration measurements in the main text. We consider here the transduction of a coherent state $|\beta(t)\rangle = |\beta_0\rangle e^{-i\Omega t}$ so that we may use the expectation values $\langle\hat{b}\rangle = \beta$ and $\langle\hat{a}\rangle = \alpha$ of the mechanical and optical fields, respectively. The equation of motion of the mechanical oscillator is given by [7]

$$\dot{\beta} = -\left(i\Omega_{\mathrm{m}}+\frac{\Gamma_{\mathrm{m}}}{2}\right)\beta + ig_0\alpha^*\alpha - \sqrt{\Gamma_{\mathrm{ex}}}\beta_{\mathrm{in}}. \tag{22}$$

In the case of low cooperativity, the second term on the right describing the optomechanical coupling can be neglected, and Eq. 22 becomes

$$\dot{\beta} = -\left(i\Omega_{\mathrm{m}}+\frac{\Gamma_{\mathrm{m}}}{2}\right)\beta - \sqrt{\Gamma_{\mathrm{ex}}}\beta_{\mathrm{in}}, \tag{23}$$

where $\beta_{\mathrm{in}} = \frac{|V_0|}{\sqrt{2Z_0\hbar\Omega}}e^{-i\Omega t}$ is the input field at the microwave port with amplitude $|V_0|$. Here, β_{in} is normalized so that $|\beta_{\mathrm{in}}|^2$ is the power carried by the incident wave in units of microwave photons per second. We solve this equation to find

$$\beta = -\chi_{\mathrm{m}}(\Omega)\sqrt{\Gamma_{\mathrm{ex}}}\beta_{\mathrm{in}} \tag{24}$$

with the mechanical susceptibility

$$\chi_{\mathrm{m}}(\Omega) = \frac{1}{\frac{\Gamma_{\mathrm{m}}}{2} - i(\Omega-\Omega_{\mathrm{m}})}. \tag{25}$$

For the optical input, we consider a coherent pump laser field $\alpha_{\mathrm{in}} = \alpha_{\mathrm{in},0}e^{-i\omega_{\mathrm{L}}t}$. In a reference frame rotating at the frequency of the pump laser, $\alpha_{\mathrm{in}} = \alpha_{\mathrm{in},0}e^{-i\omega_{\mathrm{L}}t} \mapsto \alpha_{\mathrm{in}} = \alpha_{\mathrm{in},0}$, and the equation of motion for the optical cavity field α is [7]

$$\dot{\alpha} = \left(i\Delta - \frac{\kappa}{2}\right)\alpha + ig(\beta+\beta^*) - \sqrt{\kappa_{\mathrm{ex}}}\alpha_{\mathrm{in}}, \tag{26}$$

where $\Delta = \omega_{\mathrm{L}} - \omega_{\mathrm{cav}}$ is the detuning of the laser with respect to the cavity frequency ω_{cav}, κ is the cavity decay rate, and κ_{ex} is the external coupling rate for the input laser field. The solution is

$$\alpha = -\chi_{\mathrm{o}}(0)\sqrt{\kappa_{\mathrm{ex}}}\alpha_{\mathrm{in}} + ig\chi_{\mathrm{o}}(\Omega)\beta + ig\chi_{\mathrm{o}}(-\Omega)\beta^*, \tag{27}$$

where

$$\chi_{\mathrm{o}}(\omega) = \frac{1}{\frac{\kappa}{2} - i(\Delta+\omega)} \tag{28}$$

is the frequency response of the cavity. The field-enhanced optomechanical coupling rate g is described by

$$g = g_0\bar{\alpha} = -g_0\chi_{\mathrm{o}}(0)\sqrt{\kappa_{\mathrm{ex}}}\alpha_{\mathrm{in}}. \tag{29}$$

Here, we have employed a linearized coupling interaction with $\bar{\alpha}$ as the average coherent amplitude of the optical field in the cavity. We also assume that the pump intensity is low enough that dynamical backaction can be neglected. The coherent oscillations of the mechanical oscillator with amplitude β merely lead to a frequency modulation of the optical cavity field.

Using $\beta_{\mathrm{in}} = \frac{|V_0|}{\sqrt{2Z_0\hbar\Omega}}e^{-i\Omega t}$, the expression for the intracavity field becomes

$$\alpha = -\sqrt{\kappa_{\mathrm{ex}}}\alpha_{\mathrm{in}}\chi_{\mathrm{o}}(0)\Big(1 + ig_0\chi_{\mathrm{m}}(\Omega)\sqrt{\Gamma_{\mathrm{ex}}}\frac{|V_0|}{\sqrt{2Z_0\hbar\Omega}}\chi_{\mathrm{o}}(\Omega)e^{-i\Omega t} + ig_0\chi_{\mathrm{m}}^*(\Omega)\sqrt{\Gamma_{\mathrm{ex}}}\frac{|V_0|}{\sqrt{2Z_0\hbar\Omega}}\chi_{\mathrm{o}}(-\Omega)e^{i\Omega t}\Big), \tag{30}$$

where the cavity response at the laser frequency ω_L is $\chi_\mathrm{o}(0)$, and the response at the upper and lower sideband frequencies is $\chi_\mathrm{o}(\Omega)$ and $\chi_\mathrm{o}(-\Omega)$, respectively. The cavity output field can be obtained via the relation

$$\alpha_\mathrm{out} = \alpha_\mathrm{in} + \sqrt{\kappa_\mathrm{ex}}\alpha \tag{31}$$

as

$$\begin{aligned}\alpha_\mathrm{out} = \alpha_\mathrm{in}\Big(1 &- \kappa_\mathrm{ex}\chi_\mathrm{o}(0) \\ &- i\kappa_\mathrm{ex}g_0\sqrt{\Gamma_\mathrm{ex}}\frac{|V_0|}{\sqrt{2Z_0\hbar\Omega}}\chi_\mathrm{o}(0) \\ &\cdot \big(\chi_\mathrm{m}(\Omega)\chi_\mathrm{o}(\Omega)e^{-i\Omega t} + \chi^*_\mathrm{m}(\Omega)\chi_\mathrm{o}(-\Omega)e^{i\Omega t}\big)\Big).\end{aligned} \tag{32}$$

For the sake of brevity we write

$$\alpha_\mathrm{out} = \alpha_\mathrm{in}\left(A_0 - A_-e^{-i\Omega t} - A_+e^{i\Omega t}\right), \tag{33}$$

where

$$\begin{aligned}A_0 &= 1 - \kappa_\mathrm{ex}\chi_\mathrm{o}(0) \\ A_+ &= i\kappa_\mathrm{ex}g_0\sqrt{\Gamma_\mathrm{ex}}\frac{|V_0|}{\sqrt{2Z_0\hbar\Omega}}\chi_\mathrm{o}(0)\chi_\mathrm{o}(-\Omega)\chi^*_\mathrm{m}(\Omega) \\ A_- &= i\kappa_\mathrm{ex}g_0\sqrt{\Gamma_\mathrm{ex}}\frac{|V_0|}{\sqrt{2Z_0\hbar\Omega}}\chi_\mathrm{o}(0)\chi_\mathrm{o}(\Omega)\chi_\mathrm{m}(\Omega).\end{aligned} \tag{34}$$

In our measurements, we employ a direct optical detection scheme, where the cavity output field is amplified and sent to a photoreceiver, which generates an output voltage

$$V_\mathrm{out} = -R_f\eta e r_\mathrm{phot}. \tag{35}$$

Here, R_f is the resistance of the feedback resistor of the transimpedance amplifier, η is the detector's quantum efficiency, e is the elementary charge, and $r_\mathrm{phot} = \gamma_\mathrm{opt}|\alpha_\mathrm{out}|^2$ is the incident photon flux, with γ_opt describing the overall optical gain between the cavity's output mirror and the photoreceiver. The result is

$$\begin{aligned}|\alpha_\mathrm{out}|^2 &= |\alpha_\mathrm{in}|^2|A_0 - A_-e^{-i\Omega t} - A_+e^{i\Omega t}|^2 \\ &= |\alpha_\mathrm{in}|^2\Big(|A_0|^2 + |A_-|^2 + |A_+|^2 \\ &\quad - \big(A_0A^*_- + A^*_0A_+\big)e^{i\Omega t} \\ &\quad - \big(A_0A^*_+ + A^*_0A_-\big)e^{-i\Omega t} \\ &\quad + A_-A^*_+e^{-2i\Omega t} + A^*_-A_+e^{2i\Omega t}\Big).\end{aligned} \tag{36}$$

We detect microwave-to-optical transmission with a vector network analyzer (VNA) via the scattering parameter

$$S_{21} = \gamma_\mathrm{mw}\frac{V_\mathrm{out}}{V_\mathrm{in}} \tag{37}$$

between the input field at the microwave port of the device (port 1) and the output of the photoreceiver (port 2), where γ_mw is the cable attenuation between the photoreceiver and the VNA. The VNA mixes the received signal with a local oscillator at Ω and low-pass filters the mixed signal with a detection bandwidth of 10 kHz. Consequently, the only terms of $|\alpha_\mathrm{out}|^2$ that are registered in S_{21} are those with the time dependence $e^{-i\Omega t}$. We can therefore write

$$S_{21} = \frac{1}{V_0}\gamma_\mathrm{mw}R_f\eta e\gamma_\mathrm{opt}|\alpha_\mathrm{in,0}|^2\big(A_0A^*_+ + A^*_0A_-\big). \tag{38}$$

We collect all terms that correspond to attenuation and gain in the detection chain as well as the optical power in a global detection gain factor

$$\Gamma_\mathrm{D} = \gamma_\mathrm{mw}R_f\eta e\gamma_\mathrm{opt}|\alpha_\mathrm{in,0}|^2, \tag{39}$$

which has the dimension of voltage and is calibrated in a separate measurement. We also redefine $A_\pm/V_0 \mapsto A_\pm$ such that

$$S_{21} = \Gamma_\mathrm{D}\big(A_0A^*_+ + A^*_0A_-\big)e^{-i(\Omega\tau+\theta)}, \tag{40}$$

where

$$\begin{aligned}A_+ &= i\kappa_\mathrm{ex}g_0\sqrt{\Gamma_\mathrm{ex}}\frac{1}{\sqrt{2Z_0\hbar\Omega}}\chi_\mathrm{o}(0)\chi_\mathrm{o}(-\Omega)\chi^*_\mathrm{m}(\Omega) \\ A_- &= i\kappa_\mathrm{ex}g_0\sqrt{\Gamma_\mathrm{ex}}\frac{1}{\sqrt{2Z_0\hbar\Omega}}\chi_\mathrm{o}(0)\chi_\mathrm{o}(\Omega)\chi_\mathrm{m}(\Omega).\end{aligned} \tag{41}$$

The phase offset $\phi = \Omega\tau + \theta$ accounts for the overall electronic and optical detection delay. We may also write explicitly

$$\begin{aligned}S_{21} = \frac{\Gamma_\mathrm{D}}{\sqrt{2Z_0\hbar\Omega}}g_0\sqrt{\Gamma_\mathrm{ex}}\chi_\mathrm{m}(\Omega)i\kappa_\mathrm{ex}\Big(&\big(1-\kappa_\mathrm{ex}\chi^*_\mathrm{o}(0)\big)\chi_\mathrm{o}(0)\chi_\mathrm{o}(\Omega) \\ &- \big(1-\kappa_\mathrm{ex}\chi_\mathrm{o}(0)\big)\chi^*_\mathrm{o}(0)\chi^*_\mathrm{o}(-\Omega)\Big)e^{-i(\Omega\tau+\theta)}.\end{aligned} \tag{42}$$

Finally, we separate the contribution in Eq. 42 corresponding to the electro-optomechanical response of the device,

$$\chi_\mathrm{eom}(\Omega) = g_0\sqrt{\Gamma_\mathrm{ex}}\chi_\mathrm{m}(\Omega), \tag{43}$$

from that which transduces the optomechanical frequency modulation into the detected beat-note signal,

$$\begin{aligned}\Theta(\Omega) = i\kappa_\mathrm{ex}\Big(&\big(1-\kappa_\mathrm{ex}\chi^*_\mathrm{o}(0)\big)\chi_\mathrm{o}(0)\chi_\mathrm{o}(\Omega) \\ &- \big(1-\kappa_\mathrm{ex}\chi_\mathrm{o}(0)\big)\chi^*_\mathrm{o}(0)\chi^*_\mathrm{o}(-\Omega)\Big),\end{aligned} \tag{44}$$

to obtain the more readable form

$$S_{21}(\Omega) = \frac{\Gamma_\mathrm{D}}{\sqrt{2Z_0\hbar\Omega}}\chi_\mathrm{eom}(\Omega)\Theta(\Omega)e^{-i(\Omega\tau+\theta)}. \tag{45}$$

In this form, the model can be easily expanded to include multiple (uncoupled) mechanical modes by summing their contributions to the total optical output field, giving

$$\chi_{\mathrm{eom}}(\Omega) = \sum_n g_{0,n}\sqrt{\Gamma_{\mathrm{ex},n}}\chi_{\mathrm{m},n}(\Omega)e^{i\phi_{\mathrm{m},n}}, \tag{46}$$

where an additional, mode-dependent phase offset $\phi_{\mathrm{m}} \in \{0, \pi\}$ is included to account for the relative phase difference of the mechanical displacement field between the locations along the nanobeam where coupling to the microwave and optical fields occur.

SUPPLEMENTARY NOTE 10 – CALIBRATION OF MICROWAVE-TO-OPTICAL TRANSDUCTION

A calibrated measurement of the microwave-to-optical transduction and, in particular, the electromechanical coupling rate to the transmission line, Γ_{ex}, relies on the determination of both Γ_{D} and the vacuum optomechanical coupling rate g_0. The latter is determined with a noise calibration measurement (see main text), whereas the former is ascertained from a separate measurement of the cavity response with a phase-modulated input signal. We start with the optical field from the laser, $\alpha_{\mathrm{in}} = \alpha_{\mathrm{in},0}e^{-i\omega_L t}$. For small modulation depth ϕ_0, and again switching to a frame rotating with the laser frequency, we can write the modulated optical input field as

$$\alpha_{\mathrm{in,mod}} \approx \alpha_{\mathrm{in}}\left(1 - \frac{i}{2}\phi_0\left(e^{-i\Omega t} + e^{i\Omega t}\right)\right). \tag{47}$$

For a modulation frequency Ω far from a mechanical resonance, there will be no significant optomechnical coupling, and the intracavity field can then be obtained as

$$\alpha = -\sqrt{\kappa_{\mathrm{ex}}}\alpha_{\mathrm{in}}\Big(\chi_{\mathrm{o}}(0) - \frac{i}{2}\phi_0\left(\chi_{\mathrm{o}}(\Omega)e^{-i\Omega t} + \chi_{\mathrm{o}}(-\Omega)e^{i\Omega t}\right)\Big). \tag{48}$$

Using Eq. 31, the output field is

$$\alpha_{\mathrm{out}} = \alpha_{\mathrm{in}}\left(A_0 - A_- e^{-i\Omega t} - A_+ e^{i\Omega t}\right), \tag{49}$$

where

$$\begin{aligned} A_0 &= 1 - \kappa_{\mathrm{ex}}\chi_{\mathrm{o}}(0) \\ A_+ &= \frac{i}{2}\phi_0\left(1 - \kappa_{\mathrm{ex}}\chi_{\mathrm{o}}(-\Omega)\right) \\ A_- &= \frac{i}{2}\phi_0\left(1 - \kappa_{\mathrm{ex}}\chi_{\mathrm{o}}(\Omega)\right). \end{aligned} \tag{50}$$

The modulation depth ϕ_0 as a function of input voltage amplitude V_0 is given by

$$\phi_0 = \frac{V_0}{V_\pi}\pi, \tag{51}$$

where V_π is the half-wave voltage of the electro-optic phase modulator. The cavity response is measured via the scattering parameter S_{23} in a similar fashion to the measurement of S_{21} discussed above, with the phase modulator connected to port 3 of the VNA. We can write S_{23} in a way analogous to Eq. 40 as

$$S_{23} = \Gamma_{\mathrm{D}}\left(A_0 A_+^* + A_0^* A_-\right)e^{-i(\Omega\tau+\theta)}. \tag{52}$$

where we again redefine $A_\pm/V_0 \mapsto A_\pm$ such that

$$\begin{aligned} A_+ &= \frac{i}{2}\frac{\pi}{V_\pi}\left(1 - \kappa_{\mathrm{ex}}\chi_{\mathrm{o}}(-\Omega)\right) \\ A_- &= \frac{i}{2}\frac{\pi}{V_\pi}\left(1 - \kappa_{\mathrm{ex}}\chi_{\mathrm{o}}(\Omega)\right). \end{aligned} \tag{53}$$

Importantly, we record S_{23} and S_{21} in back-to-back measurements at the same optical power, which allows us to infer Γ_{D} if the half-wave voltage V_π is known. Note that the detection delay $\phi = \Omega\tau + \theta$ here is not the same as in Eq. 40, as the cable and fiber lengths are not necessarily matched. Supplementary Fig. 10 shows a fit of Eq. 52 to the calibration measurement used for the transduction spectrum presented in Fig. 5(c) in the main text.

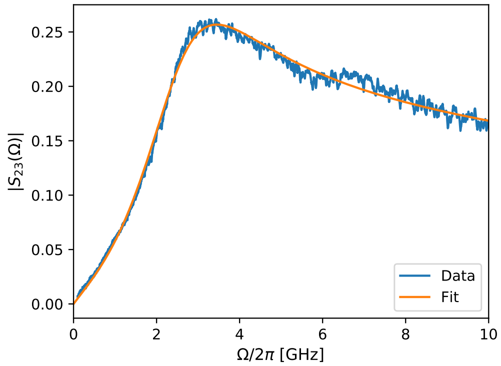

SUPPLEMENTARY FIG. 10. **Cavity response calibration**. Cavity response measured by VNA (blue) and fit to Eq. 52 (orange).

SUPPLEMENTARY NOTE 11 – DETERMINATION OF V_π

We calibrate V_π as a function of the modulation frequency Ω via the ratio of the carrier power P_{car} and that of the first sideband $P_{\mathrm{sb},1}$, which is given in terms of the zeroth- and first-order Bessel functions $J_0(\phi_0)$ and $J_1(\phi_0)$ as

$$\rho_{\mathrm{sb}} = \frac{P_{\mathrm{sb},1}}{P_{\mathrm{car}}} = \frac{J_1(\phi_0)^2}{J_0(\phi_0)^2}. \tag{54}$$

We measure the sideband ratio using a scanning Fabry-Pérot cavity (Thorlabs SA210-12B) with 10 GHz free-spectral-range as shown in Supplementary Fig. 11. A

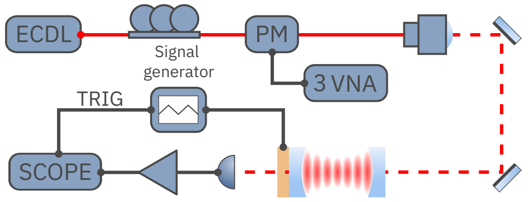

SUPPLEMENTARY FIG. 11. **Apparatus for calibration of V_π**. Light from an external-cavity diode laser (ECDL) is modulated by a phase modulator (PM) that is driven via port 3 of the VNA. The signal is then coupled to free-space and sent to a scanning Fabry-Pérot cavity. The transmitted power is detected and recorded on an oscilloscope (SCOPE).

carrier signal is produced with an external-cavity diode laser (Photonetics Tunics Plus) close to the resonance wavelength of the photonic crystal cavity (1522 nm). The carrier is modulated at various microwave frequencies using an electro-optic phase modulator (Thorlabs LN65S-FC) driven by port 3 of the VNA. The modulated signal is coupled to free-space and aligned to the Fabry-Pérot cavity. The transmitted light is detected on the built-in photodiode and amplified (Thorlabs SA201). By varying the length of the cavity continuously with a voltage ramp (Thorlabs SA201) applied to the piezo element of the cavity, we separate the carrier and sideband amplitudes. The signal is recorded on an oscilloscope. A typical time trace from the oscilloscope is shown in Supplementary Fig. 12(a) for a modulation frequency of 3 GHz. The sideband ratio is measured for various modulation depths and fit to Eq. 54 with an offset to account for the noise level. A typical result is shown in Supplementary Fig. 12(b). The finesse and free-spectral-range of the cavity allows us to record the sideband ratios for $2\,\text{GHz} \leq \Omega \leq 4\,\text{GHz}$ and $6\,\text{GHz} \leq \Omega \leq 8\,\text{GHz}$. The measured V_π as a function of the modulation frequency is shown in Supplementary Fig. 13. We approximate the frequency dependence of V_π with a linear function, which we use for the calibration of microwave-to-optical transduction described above.

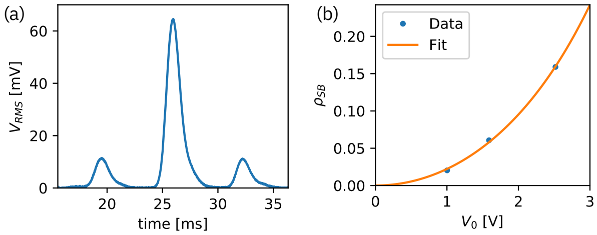

SUPPLEMENTARY FIG. 12. **V_π calibration**. (a) Typical transmission trace of the Fabry-Pérot cavity with the peak corresponding to the carrier in the center and the sidebands to the left and right at 3 GHz modulation frequency. (b) First sideband ratios recorded at various input voltages V_0 (blue) and fit to Eq. 54 (orange).

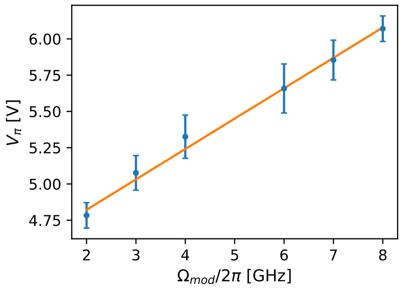

SUPPLEMENTARY FIG. 13. **Frequency-dependence of V_π**. Values of V_π extracted at various modulation frequencies (blue) and linear fit (orange).

SUPPLEMENTARY NOTE 12 – NONLINEAR ELECTROMECHANICAL RESPONSE

Linear coherent transduction is shown in the main text with a Gaussian noise distribution of the transduced

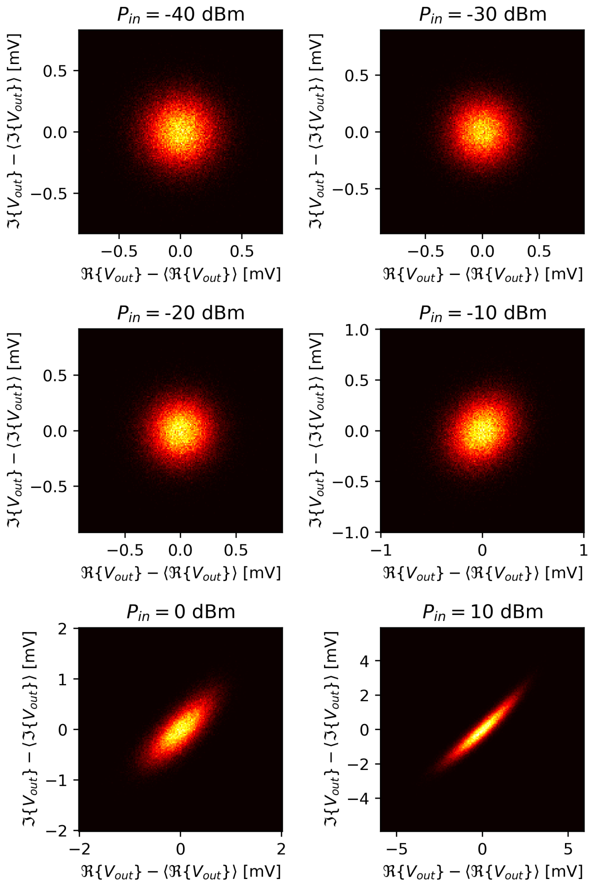

SUPPLEMENTARY FIG. 14. **Noise distribution of the transduced coherent tone for various drive powers**. Noise distribution recorded from the time trace of transduced continuous-wave signals for various microwave input powers. For $P_{\text{in}} \leq -20\,\text{dBm}$, the distribution is Gaussian. At higher powers, the noise increases and is correlated between quadratures.

tone. We note here that the device shows interesting noise behavior at higher microwave input power. The noise distribution of the transduced tone is shown in Supplementary Fig. 14 for microwave input powers spanning five orders of magnitude. The distribution is Gaussian with a relatively constant variance for microwave input levels $\leq -20\,\mathrm{dBm}$. At higher powers, we observe increasingly non-Gaussian noise in the transduced tone. We attribute the anisotropy in the noise to a nonlinear response of the mechanical oscillator that maps the coherent microwave input tone to a non-circular phase-space trajectory. The mean output voltage of the transduced signal as recorded by the VNA as a function of input voltage is shown in Supplementary Fig. 15(a). We note that the amplitude of the transduced signal remains linear over most of the observed range. The slight deviation from linearity for the lowest input power may be due to noise in our measurement apparatus. The noise variance along the real axis is shown in Supplementary Fig. 15(b) as a function of the phase angle ϕ.

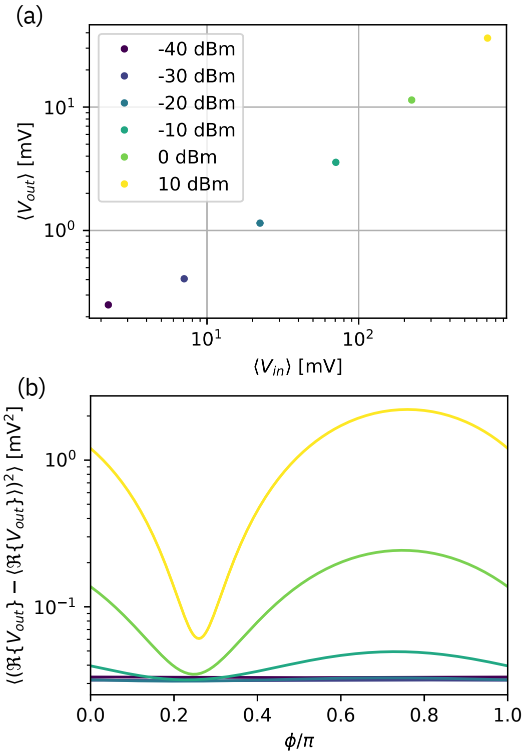

SUPPLEMENTARY FIG. 15. **(a) Characteristics of transduced tone at various input powers**. Mean displacement of transduced state for various microwave input powers. (b) Anisotropy of transduced noise measured along real axis as a function of the signal phase ϕ.

* pfs@zurich.ibm.com

[1] Schneider, K. *et al.* Gallium phosphide-on-silicon dioxide photonic devices. *Journal of Lightwave Technology* **36**, 2994–3002 (2018).

[2] Hönl, S., Hahn, H., Baumgartner, Y., Czornomaz, L. & Seidler, P. Highly selective dry etching of GaP in the presence of $Al_xGa_{1-x}P$ with a $SiCl_4/SF_6$ plasma. *Journal of Physics D: Applied Physics* **51**, 185203 (2018).

[3] Wilson, D. J. *et al.* Integrated gallium phosphide nonlinear photonics. *Nature Photonics* **14**, 57–62 (2020).

[4] Burek, M. J. *et al.* Fiber-coupled diamond quantum nanophotonic interface. *Physical Review Applied* **8**, 024026 (2017).

[5] Girvin, S. M. Circuit QED: superconducting qubits coupled to microwave photons. *Quantum machines: measurement and control of engineered quantum systems* **113**, 2 (2011).

[6] Pozar, D. M. *Microwave engineering* (John Wiley & Sons, 2011).

[7] Aspelmeyer, M., Kippenberg, T. J. & Marquardt, F. Cavity optomechanics. *Reviews of Modern Physics* **86**, 1391 (2014).
